# The genome sequence of the Stripe-backed Dasysyrphus, *Dasysyrphus albostriatus *(Fallén, 1817)

**DOI:** 10.12688/wellcomeopenres.20887.1

**Published:** 2024-02-15

**Authors:** Liam M. Crowley, Denise C. Wawman, University of Oxford and Wytham Woods Genome Acquisition Lab, Darwin Tree of Life Barcoding collective, Wellcome Sanger Institute Tree of Life Management, Samples and Laboratory team, Wellcome Sanger Institute Scientific Operations: Sequencing Operations, Wellcome Sanger Institute Tree of Life Core Informatics team, Tree of Life Core Informatics collective, Darwin Tree of Life Consortium

**Affiliations:** 1 Department of Biology, University of Oxford, Oxford, England, UK

**Keywords:** Dasysyrphus albostriatus, stripe-backed Dasysyrphus, genome sequence, chromosomal, Diptera

## Abstract

We present a genome assembly from an individual female *Dasysyrphus albostriatus* (the Stripe-backed Dasysyrphus; Arthropoda; Insecta; Diptera; Syrphidae). The genome sequence is 662.5 megabases in span. Most of the assembly is scaffolded into 5 chromosomal pseudomolecules, including the X sex chromosome. The mitochondrial genome has also been assembled and is 17.55 kilobases in length. Gene annotation of this assembly on Ensembl identified 12,259 protein coding genes.

## Species taxonomy

Eukaryota; Opisthokonta; Metazoa; Eumetazoa; Bilateria; Protostomia; Ecdysozoa; Panarthropoda; Arthropoda; Mandibulata; Pancrustacea; Hexapoda; Insecta; Dicondylia; Pterygota; Neoptera; Endopterygota; Diptera; Brachycera; Muscomorpha; Eremoneura; Cyclorrhapha; Aschiza; Syrphoidea; Syrphidae; Syrphinae; Syrphini; *Dasysyrphus; Dasysyrphus albostriatus* (Fallén, 1817) (NCBI:txid414801).

## Background

The Stripe-backed Dasysyrphus, *Dasysyrphus albostriatus*, is a medium-sized black and yellow striped hoverfly in the subfamily Syrphinae. It occurs across the Palearctic and is widespread throughout Great Britain and Ireland in woodland clearings and edges ( Stubbs & Falk, 2002). *Dasysyrphus* species can be distinguished from hoverflies in other genera by hairy eyes and a long black wing stigma, a dark marking along the leading edge of the wing. *Dasysyrphus albostriatus* has yellow bars on the second, third and fourth abdominal segments that are angled downwards and often fused to form an inverted ‘v’ ( Ball & Morris, 2013). There are a pair of grey stripes on the thorax, from which the common name is derived.

It is bivoltine, with the first flight period from April to June and a second August to September ( Ball & Morris, 2013). Both sexes visit a wide range of flowers. The larvae are aphidophagous, feeding on a range of arboreal aphid species at night and resting in the trees near the aphid colony during the day, protected by their camouflage, of dark colours and projections from their bodies, that helps them to blend in with twigs and the bark of branches ( Hoverfly Recording Scheme, 2022).

We present a chromosomally complete genome sequence for *Dasysyrphus albostriatus* based on one adult female specimen, sequenced as part of the Darwin Tree of Life Project. This project is a collaborative effort to sequence all named eukaryotic species in the Atlantic Archipelago of Britain and Ireland.

## Genome sequence report

**Figure 1.**
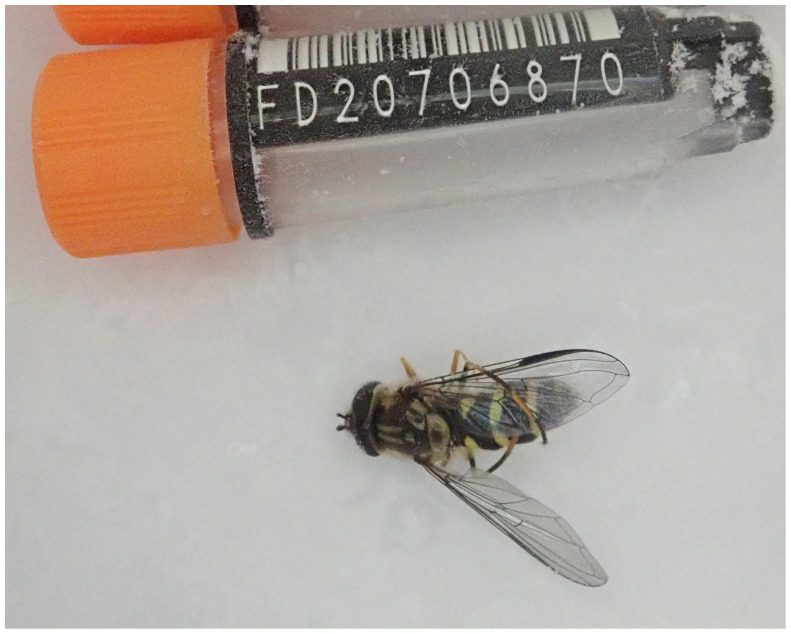
Photograph of the *Dasysyrphus albostriatus* (idDasAlbo1) specimen used for genome sequencing.

The genome was sequenced from one female *Dasysyrphus albostriatus* ( Figure 1) collected from Tubney House, Oxfordshire, UK (51.69, –1.37). A total of 29-fold coverage in Pacific Biosciences single-molecule HiFi long reads was generated. Primary assembly contigs were scaffolded with chromosome conformation Hi-C data. Manual assembly curation corrected 130 missing joins or mis-joins and removed 15 haplotypic duplications, reducing the assembly length by 1.35% and the scaffold number by 72.15%.

**Figure 2.**
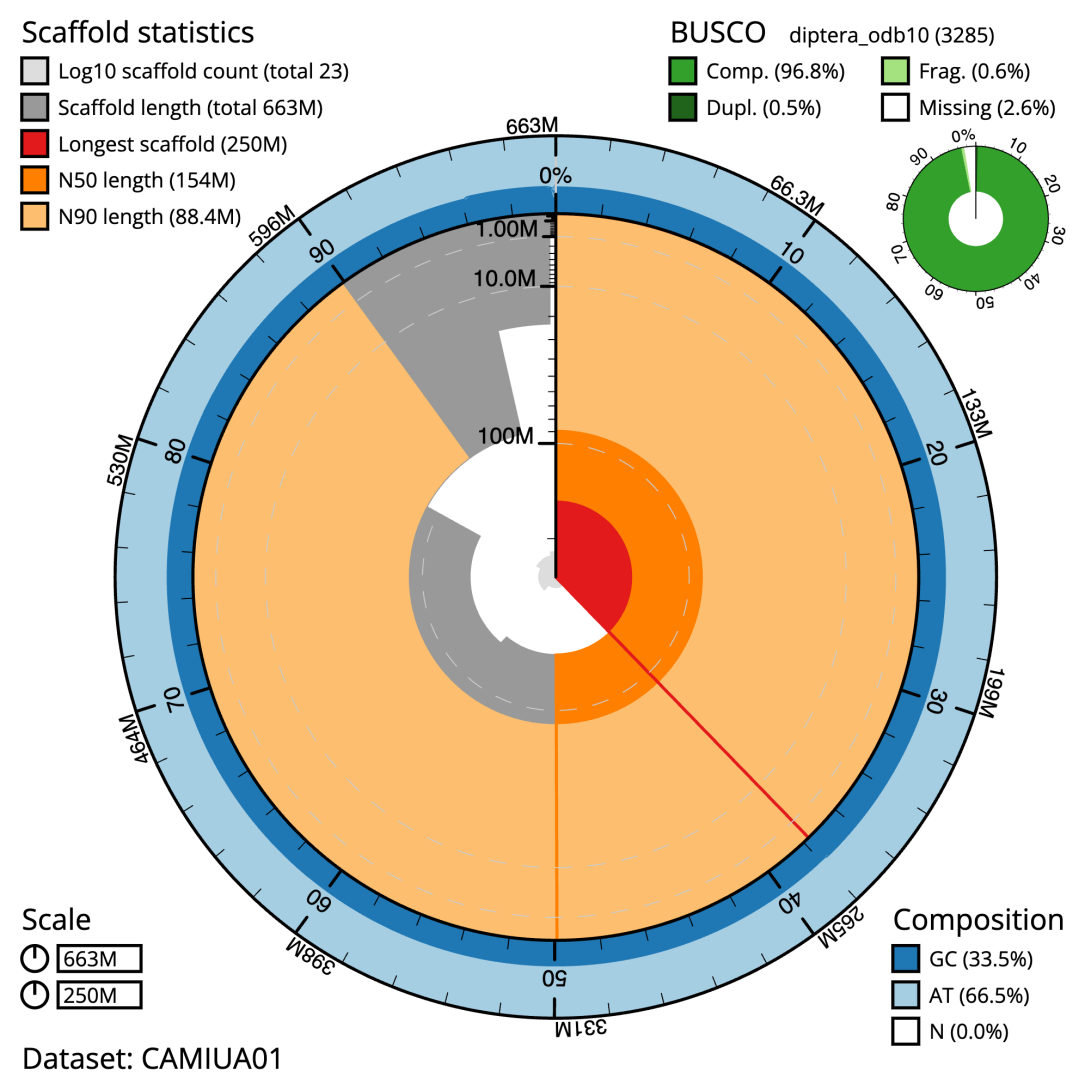
Genome assembly of *Dasysyrphus albostriatus*, idDasAlbo1.1: metrics. The BlobToolKit Snailplot shows N50 metrics and BUSCO gene completeness. The main plot is divided into 1,000 size-ordered bins around the circumference with each bin representing 0.1% of the 662,557,047 bp assembly. The distribution of scaffold lengths is shown in dark grey with the plot radius scaled to the longest scaffold present in the assembly (249,865,697 bp, shown in red). Orange and pale-orange arcs show the N50 and N90 scaffold lengths (154,042,049 and 88,412,347 bp), respectively. The pale grey spiral shows the cumulative scaffold count on a log scale with white scale lines showing successive orders of magnitude. The blue and pale-blue area around the outside of the plot shows the distribution of GC, AT and N percentages in the same bins as the inner plot. A summary of complete, fragmented, duplicated and missing BUSCO genes in the diptera_odb10 set is shown in the top right. An interactive version of this figure is available at https://blobtoolkit.genomehubs.org/view/CAMIUA01/dataset/CAMIUA01/snail.

**Figure 3.**
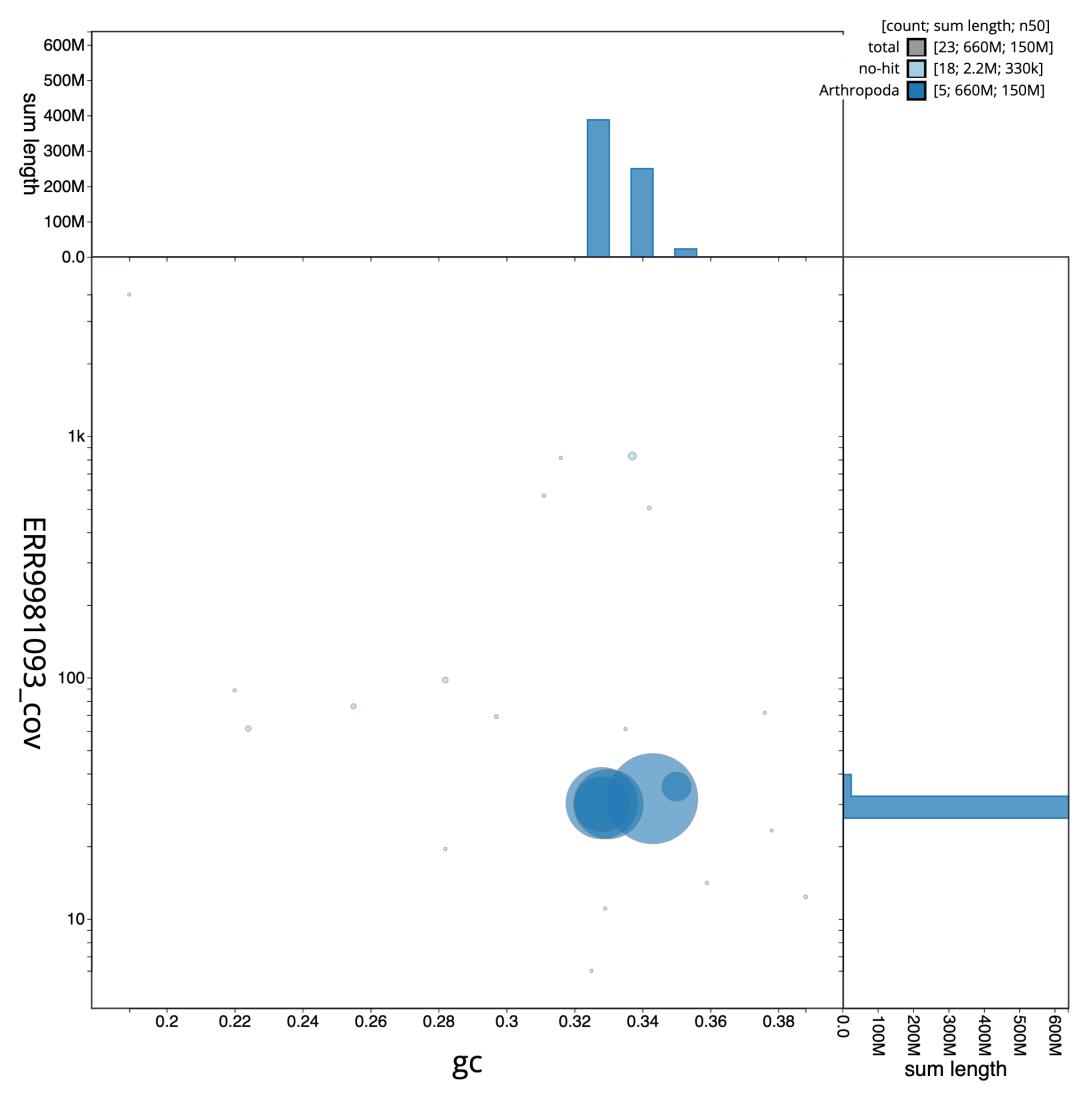
Genome assembly of *Dasysyrphus albostriatus*, idDasAlbo1.1: BlobToolKit GC-coverage plot. Scaffolds are coloured by phylum. Circles are sized in proportion to scaffold length. Histograms show the distribution of scaffold length sum along each axis. An interactive version of this figure is available at https://blobtoolkit.genomehubs.org/view/CAMIUA01/dataset/CAMIUA01/blob.

**Figure 4.**
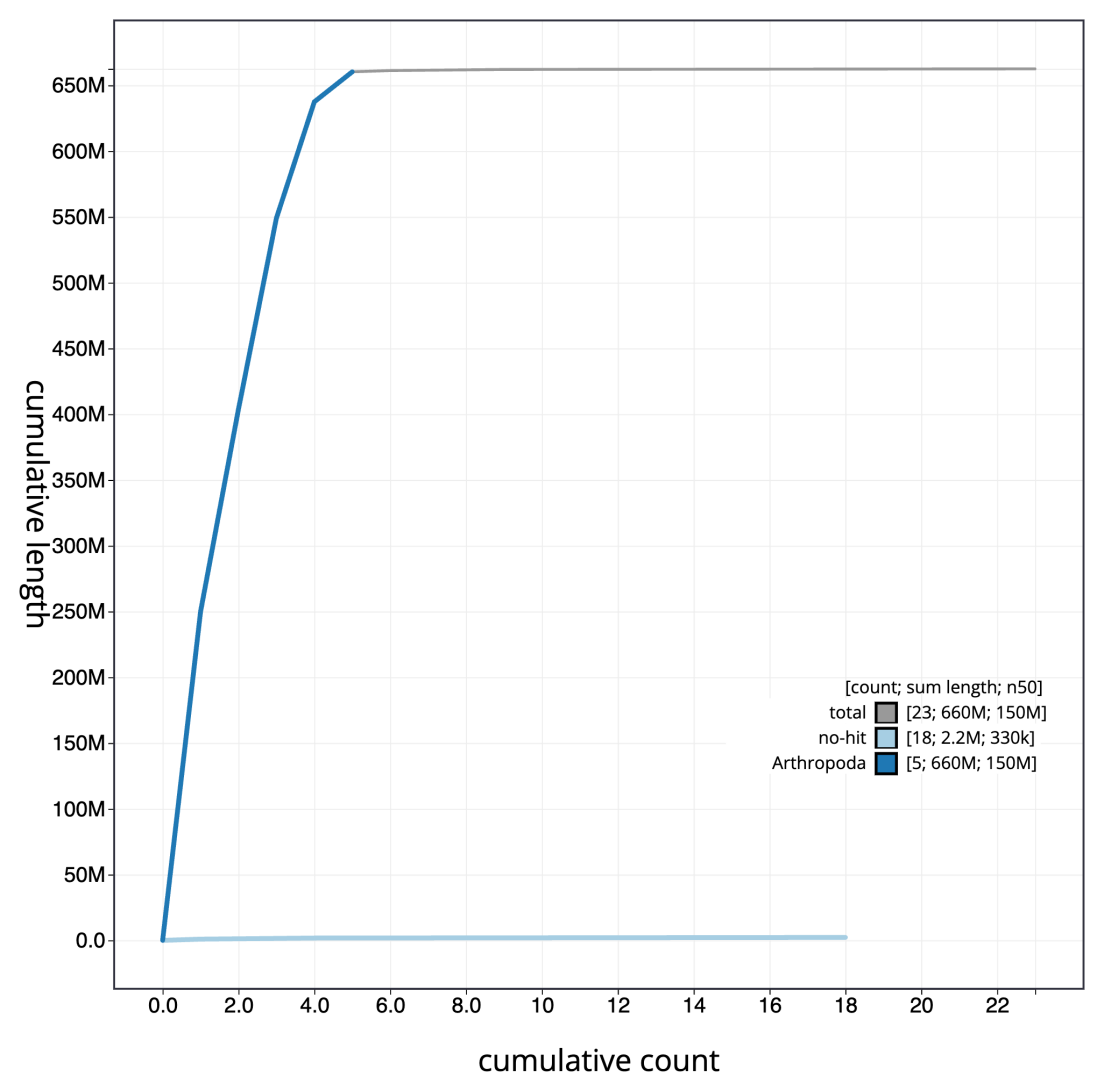
Genome assembly of *Dasysyrphus albostriatus*, idDasAlbo1.1: BlobToolKit cumulative sequence plot. The grey line shows cumulative length for all scaffolds. Coloured lines show cumulative lengths of scaffolds assigned to each phylum using the buscogenes taxrule. An interactive version of this figure is available at https://blobtoolkit.genomehubs.org/view/CAMIUA01/dataset/CAMIUA01/cumulative.

**Figure 5.**
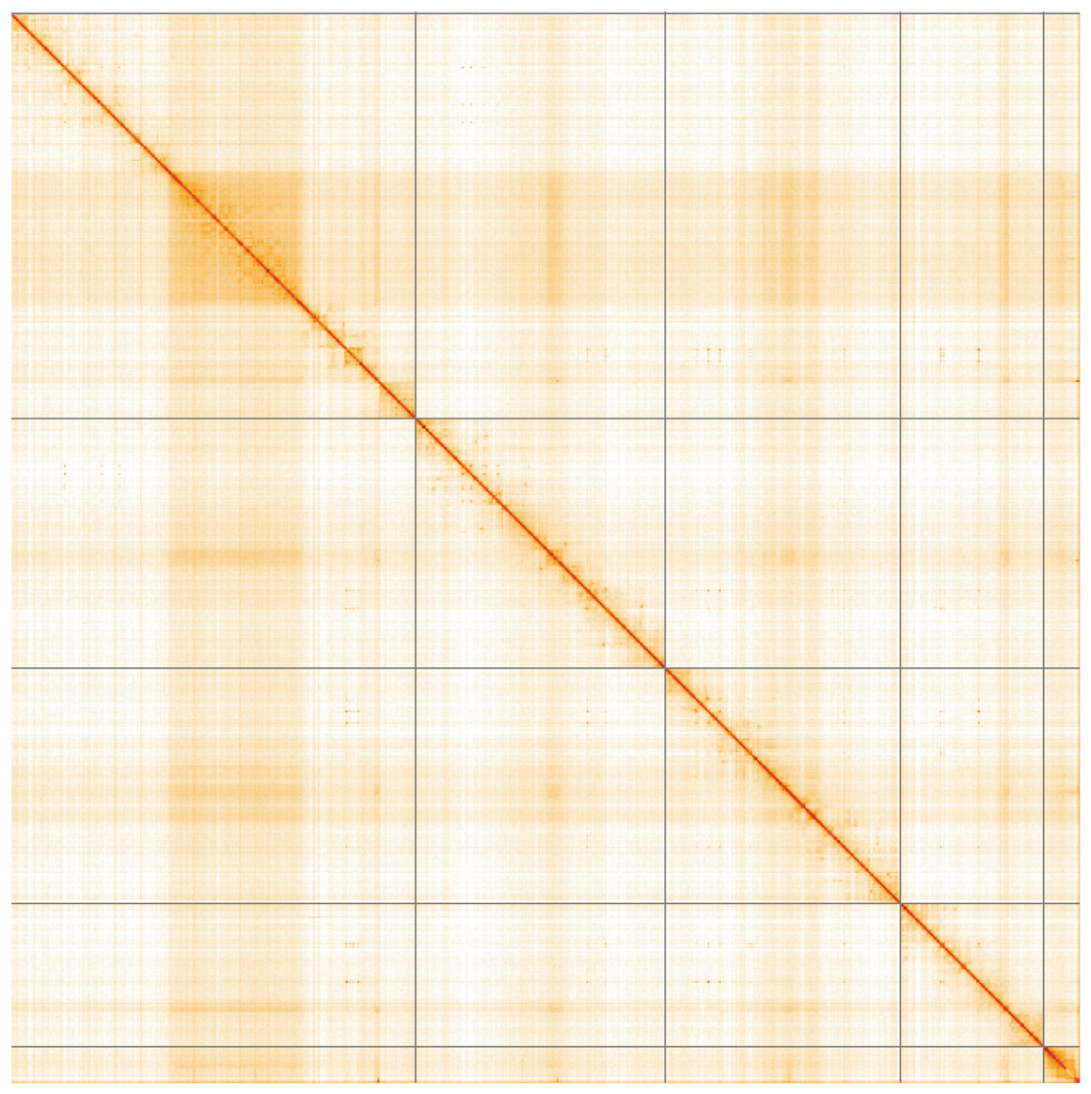
Genome assembly of *Dasysyrphus albostriatus*, idDasAlbo1.1: Hi-C contact map of the idDasAlbo1.1 assembly, visualised using HiGlass. Chromosomes are shown in order of size from left to right and top to bottom. An interactive version of this figure may be viewed at https://genome-note-higlass.tol.sanger.ac.uk/l/?d=G3xi3rX8S4y1rbDptjgNRw.

The final assembly has a total length of 662.5 Mb in 22 sequence scaffolds with a scaffold N50 of 154.0 Mb ( Table 1). The snailplot in Figure 2 provides a summary of the assembly statistics, while the distribution of assembly scaffolds on GC proportion and coverage is shown in Figure 3. The cumulative assembly plot in Figure 4 shows curves for subsets of scaffolds assigned to different phyla. Most (99.98%) of the assembly sequence was assigned to 5 chromosomal-level scaffolds, representing 4 autosomes and the X sex chromosome. Chromosome-scale scaffolds confirmed by the Hi-C data are named in order of size ( Figure 5; Table 2). The scaffold order and orientation uncertain on chromosome X in the region of 15.31–18.96 Mb. While not fully phased, the assembly deposited is of one haplotype. Contigs corresponding to the second haplotype have also been deposited. The mitochondrial genome was also assembled and can be found as a contig within the multifasta file of the genome submission.

**Table 1.** Genome data for *Dasysyrphus albostriatus*, idDasAlbo1.1.

Project accession data		
Assembly identifier	idDasAlbo1.1	
Species	*Dasysyrphus albostriatus*	
Specimen	idDasAlbo1	
NCBI taxonomy ID	414801	
BioProject	PRJEB54801	
BioSample ID	SAMEA10166817	
Isolate information	idDasAlbo1, female: thorax (DNA sequencing), head (Hi-C sequencing), abdomen (RNA sequencing)	
Assembly metrics *		*Benchmark*
Consensus quality (QV)	62.5	*≥ 50*
*k*-mer completeness	100.0%	*≥ 95%*
BUSCO **	C:96.8%[S:96.3%,D:0.5%], F:0.6%,M:2.6%,n:3,285	*C ≥ 95%*
Percentage of assembly mapped to chromosomes	99.98%	*≥ 95%*
Sex chromosomes	XX	*localised * *homologous pairs*
Organelles	Mitochondrial genome: 17.55 kb	*complete single * *alleles*
Raw data accessions		
PacificBiosciences SEQUEL II	ERR9981093	
Hi-C Illumina	ERR9988135	
PolyA RNA-Seq Illumina	ERR10378022	
Genome assembly		
Assembly accession	GCA_946251815.1	
*Accession of alternate * *haplotype*	GCA_946251825.1	
Span (Mb)	662.5	
Number of contigs	287	
Contig N50 length (Mb)	6.4	
Number of scaffolds	22	
Scaffold N50 length (Mb)	154.0	
Longest scaffold (Mb)	249.87	
Genome annotation		
Number of protein-coding genes	12,259	
Number of non-coding genes	1,527	
Number of gene transcripts	20,312	

* Assembly metric benchmarks are adapted from column VGP-2020 of “Table 1: Proposed standards and metrics for defining genome assembly quality” from Rhie *et al.* (2021). ** BUSCO scores based on the diptera_odb10 BUSCO set using version 5.3.2. C = complete [S = single copy, D = duplicated], F = fragmented, M = missing, n = number of orthologues in comparison. A full set of BUSCO scores is available at https://blobtoolkit.genomehubs.org/view/CAMIUA01/dataset/CAMIUA01/busco.

**Table 2.** Chromosomal pseudomolecules in the genome assembly of Dasysyrphus albostriatus, idDasAlbo1.

INSDC accession	Chromosome	Length (Mb)	GC%
OX276335.1	1	249.87	34.5
OX276336.1	2	154.04	33.0
OX276337.1	3	145.18	33.0
OX276338.1	4	88.41	33.0
OX276339.1	X	22.82	35.0
OX276340.1	MT	0.02	19.0

The estimated Quality Value (QV) of the final assembly is 62.5 with *k*-mer completeness of 100.0%, and the assembly has a BUSCO v5.3.2 completeness of 96.8% (single = 96.3%, duplicated = 0.5%), using the diptera_odb10 reference set ( *n* = 3,285).

Metadata for specimens, barcode results, spectra estimates, sequencing runs, contaminants and pre-curation assembly statistics are given at https://links.tol.sanger.ac.uk/species/414801.

## Genome annotation report

The *Dasysyrphus albostriatus* genome assembly (GCA_946251815.1) was annotated using the Ensembl rapid annotation pipeline ( Table 1; https://rapid.ensembl.org/Dasysyrphus_albostriatus_GCA_946251815.1/Info/Index). The resulting annotation includes 20,312 transcribed mRNAs from 12,259 protein-coding and 1,527 non-coding genes.

## Methods

### Sample acquisition and nucleic acid extraction

A female *Dasysyrphus albostriatus* (specimen ID Ox001343, ToLID idDasAlbo1) was netted at Tubney House, Oxfordshire, UK (latitude 51.69, longitude –1.37) on 2021-05-10. The specimen was collected and identified by Liam Crowley (University of Oxford) and preserved on dry ice.

Protocols developed by the Wellcome Sanger Institute (WSI) Tree of Life core laboratory have been deposited on protocols.io ( Denton *et al.*, 2023b). The workflow for high molecular weight (HMW) DNA extraction at the WSI includes a sequence of core procedures: sample preparation; sample homogenisation, DNA extraction, fragmentation, and clean-up. In sample preparation, the idDasAlbo1 sample was weighed and dissected on dry ice ( Jay *et al.*, 2023). Tissue from the thorax was homogenised using a PowerMasher II tissue disruptor ( Denton *et al.*, 2023a).HMW DNA was extracted in the WSI Scientific Operations core using the Automated MagAttract v2 protocol ( Oatley *et al.*, 2023). HMW DNA was sheared into an average fragment size of 12–20 kb in a Megaruptor 3 system with speed setting 31 ( Bates *et al.*, 2023). Sheared DNA was purified by solid-phase reversible immobilisation ( Strickland *et al.*, 2023): in brief, the method employs a 1.8X ratio of AMPure PB beads to sample to eliminate shorter fragments and concentrate the DNA. The concentration of the sheared and purified DNA was assessed using a Nanodrop spectrophotometer and Qubit Fluorometer and Qubit dsDNA High Sensitivity Assay kit. Fragment size distribution was evaluated by running the sample on the FemtoPulse system.

RNA was extracted from abdomen tissue of idDasAlbo1 in the Tree of Life Laboratory at the WSI using the RNA Extraction: Automated MagMax™ *mir*Vana protocol ( do Amaral *et al.*, 2023). The RNA concentration was assessed using a Nanodrop spectrophotometer and a Qubit Fluorometer using the Qubit RNA Broad-Range Assay kit. Analysis of the integrity of the RNA was done using the Agilent RNA 6000 Pico Kit and Eukaryotic Total RNA assay.

### Sequencing

Pacific Biosciences HiFi circular consensus DNA sequencing libraries were constructed according to the manufacturers’ instructions. Poly(A) RNA-Seq libraries were constructed using the NEB Ultra II RNA Library Prep kit. DNA and RNA sequencing was performed by the Scientific Operations core at the WSI on Pacific Biosciences SEQUEL II (HiFi) and Illumina NovaSeq 6000 (RNA-Seq) instruments. Hi-C data were also generated from head tissue of idDasAlbo1 using the Arima2 kit and sequenced on the Illumina NovaSeq 6000 instrument.

### Genome assembly, curation and evaluation

Assembly was carried out with Hifiasm ( Cheng *et al.*, 2021) and haplotypic duplication was identified and removed with purge_dups ( Guan *et al.*, 2020). The assembly was then scaffolded with Hi-C data ( Rao *et al.*, 2014) using YaHS ( Zhou *et al.*, 2023). The assembly was checked for contamination and corrected as described previously ( Howe *et al.*, 2021). Manual curation was performed using HiGlass ( Kerpedjiev *et al.*, 2018) and Pretext ( Harry, 2022). The mitochondrial genome was assembled using MitoHiFi ( Uliano-Silva *et al.*, 2023), which runs MitoFinder ( Allio *et al.*, 2020) or MITOS ( Bernt *et al.*, 2013) and uses these annotations to select the final mitochondrial contig and to ensure the general quality of the sequence.

A Hi-C map for the final assembly was produced using bwa-mem2 ( Vasimuddin *et al.*, 2019) in the Cooler file format ( Abdennur & Mirny, 2020). To assess the assembly metrics, the *k*-mer completeness and QV consensus quality values were calculated in Merqury ( Rhie *et al.*, 2020). This work was done using Nextflow ( Di Tommaso *et al.*, 2017) DSL2 pipelines “sanger-tol/readmapping” ( Surana *et al.*, 2023a) and “sanger-tol/genomenote” ( Surana *et al.*, 2023b). The genome was analysed within the BlobToolKit environment ( Challis *et al.*, 2020) and BUSCO scores ( Manni *et al.*, 2021; Simão *et al.*, 2015) were calculated.

Table 3 contains a list of relevant software tool versions and sources.

**Table 3.** Software tools: versions and sources.

Software tool	Version	Source
BlobToolKit	4.1.7	https://github.com/blobtoolkit/blobtoolkit
BUSCO	5.3.2	https://gitlab.com/ezlab/busco
Hifiasm	0.16.1-r375	https://github.com/chhylp123/hifiasm
HiGlass	1.11.6	https://github.com/higlass/higlass
Merqury	MerquryFK	https://github.com/thegenemyers/MERQURY.FK
MitoHiFi	2	https://github.com/marcelauliano/MitoHiFi
PretextView	0.2	https://github.com/wtsi-hpag/PretextView
purge_dups	1.2.3	https://github.com/dfguan/purge_dups
sanger-tol/genomenote	v1.0	https://github.com/sanger-tol/genomenote
sanger-tol/readmapping	1.1.0	https://github.com/sanger-tol/readmapping/tree/1.1.0
YaHS	yahs-1.1.91eebc2	https://github.com/c-zhou/yahs

### Genome annotation

The Ensembl gene annotation system ( Aken *et al.*, 2016) was used to generate annotation for the *Dasysyrphus albostriatus* assembly (GCA_946251815.1). Annotation was created primarily through alignment of transcriptomic data to the genome, with gap filling via protein-to-genome alignments of a select set of proteins from UniProt ( UniProt Consortium, 2019).

### Wellcome Sanger Institute – Legal and Governance

The materials that have contributed to this genome note have been supplied by a Darwin Tree of Life Partner. The submission of materials by a Darwin Tree of Life Partner is subject to the **‘Darwin Tree of Life Project Sampling Code of Practice’**, which can be found in full on the Darwin Tree of Life website here. By agreeing with and signing up to the Sampling Code of Practice, the Darwin Tree of Life Partner agrees they will meet the legal and ethical requirements and standards set out within this document in respect of all samples acquired for, and supplied to, the Darwin Tree of Life Project.

Further, the Wellcome Sanger Institute employs a process whereby due diligence is carried out proportionate to the nature of the materials themselves, and the circumstances under which they have been/are to be collected and provided for use. The purpose of this is to address and mitigate any potential legal and/or ethical implications of receipt and use of the materials as part of the research project, and to ensure that in doing so we align with best practice wherever possible. The overarching areas of consideration are:

- Ethical review of provenance and sourcing of the material
- Legality of collection, transfer and use (national and international)

Each transfer of samples is further undertaken according to a Research Collaboration Agreement or Material Transfer Agreement entered into by the Darwin Tree of Life Partner, Genome Research Limited (operating as the Wellcome Sanger Institute), and in some circumstances other Darwin Tree of Life collaborators.

## Data Availability

European Nucleotide Archive: *Dasysyrphus albostriatus* (stripe-backed Dasysyrphus). Accession number PRJEB54801; https://identifiers.org/ena.embl/PRJEB54801 ( Wellcome Sanger Institute, 2022). The genome sequence is released openly for reuse. The *Dasysyrphus albostriatus* genome sequencing initiative is part of the Darwin Tree of Life (DToL) project. All raw sequence data and the assembly have been deposited in INSDC databases. Raw data and assembly accession identifiers are reported in Table 1.

## Author information

Members of the University of Oxford and Wytham Woods Genome Acquisition Lab are listed here: https://doi.org/10.5281/zenodo.7125292.

Members of the Darwin Tree of Life Barcoding collective are listed here: https://doi.org/10.5281/zenodo.4893703.

Members of the Wellcome Sanger Institute Tree of Life Management, Samples and Laboratory team are listed here: https://doi.org/10.5281/zenodo.10066175.

Members of Wellcome Sanger Institute Scientific Operations: Sequencing Operations are listed here: https://doi.org/10.5281/zenodo.10043364.

Members of the Wellcome Sanger Institute Tree of Life Core Informatics team are listed here: https://doi.org/10.5281/zenodo.10066637.

Members of the Tree of Life Core Informatics collective are listed here: https://doi.org/10.5281/zenodo.5013541.

Members of the Darwin Tree of Life Consortium are listed here: https://doi.org/10.5281/zenodo.4783558.
