# Supplementary material for: The genome sequence of the Stripe-backed Dasysyrphus, Dasysyrphus albostriatus (Fallén, 1817)
Source: Wellcome Open Res. 2024 Feb 15;9:34. [Version 1] doi: 10.12688/wellcomeopenres.20887.1 (PMC11310654; doi:10.12688/wellcomeopenres.20887.1)
Supplement: Peer review history

# Reviewer report: Reviewer response for version 1

**DOI:** 10.21956/wellcomeopenres.23111.r88981

**Published:** 2024-08-08

**Authors:** Halide Nihal Açıkgöz

**Affiliations:** 1 Ankara University, Ankara, Turkey

Dear Editor,

**Title**: The title clearly indicates the main subject of the study.

**Abstract**: The abstract succinctly summarizes the study's objectives, methods, and findings.

**Background Information**: The introduction provides adequate information about the biology and ecology of *Dasysyrphus albostriatus*. It explains why this species is significant for genetic studies.

**Objective and Hypothesis**: The study's objective is clearly stated, emphasizing the contribution of completing the genome sequence to biological research.

**Methods: **The methods are sufficiently detailed to allow replication by other researchers. However, some steps could benefit from more detailed explanations.

**Data Presentation:** The results are detailed and well-organized. Basic metrics such as genome size and gene count are clearly presented. Figures and tables facilitate data comprehension.

**Interpretation and Analysis: **The analysis and interpretation of the findings are comprehensive. Meaningful conclusions about the biological and genetic characteristics of the genome are drawn. However, some results could be discussed in greater dept.

**Summary and Future Work: **The conclusion summarizes the main findings and emphasizes the importance of this genome sequence for future studies. Suggestions for future research are provided.

Are sufficient details of methods and materials provided to allow replication by others?

Yes

Is the rationale for creating the dataset(s) clearly described?

Yes

Are the datasets clearly presented in a useable and accessible format?

Yes

Are the protocols appropriate and is the work technically sound?

Yes

Reviewer Expertise:

DNA, Forensic Entomology, Forensic Biology

I confirm that I have read this submission and believe that I have an appropriate level of expertise to confirm that it is of an acceptable scientific standard.

# Reviewer report: Reviewer response for version 1

**DOI:** 10.21956/wellcomeopenres.23111.r88980

**Published:** 2024-08-06

**Authors:** Joel Gibson

**Affiliations:** 1 Royal British Columbia Museum, Victoria, British Columbia, Canada

This is an excellent contribution to the ongoing effort to document the genomes of all species in Britain and Ireland. Many species of *Syrphidae *are important pollinators and insights into their genomes will be valuable in the future. The methods used to recover these molecular data are solid and the results can be easily accessed.

Are sufficient details of methods and materials provided to allow replication by others?

Yes

Is the rationale for creating the dataset(s) clearly described?

Yes

Are the datasets clearly presented in a useable and accessible format?

Yes

Are the protocols appropriate and is the work technically sound?

Yes

Reviewer Expertise:

Entomology, Diptera, morphology, molecular systematics, ecology, shoreline habitats

I confirm that I have read this submission and believe that I have an appropriate level of expertise to confirm that it is of an acceptable scientific standard.
